# Prospects for cereal self-sufficiency in sub-Saharan Africa

**DOI:** 10.1073/pnas.2423669122

**Published:** 2025-06-09

**Authors:** Martin K. van Ittersum, Seyyedmajid Alimagham, João Vasco Silva, Samuel Adjei-Nsiah, Frederick P. Baijukya, Abdullahi Bala, Regis Chikowo, Patricio Grassini, Hugo L.E. de Groot, Aphrodis Nshizirungu, Abdelkader Mahamane Soulé, Timothy B. Sulser, Godfrey Taulya, Fatima Amor Tenorio, Kindie Tesfaye, Shen Yuan, Marloes P. van Loon

**Affiliations:** ^a^Plant Production Systems Group, Wageningen University & Research, Wageningen 6700AK, The Netherlands; ^b^Department of Crop Production Ecology, Swedish University of Agricultural Sciences, Uppsala SE-75007, Sweden; ^c^Sustainable Agrifood Systems, International Maize and Wheat Improvement Center (CIMMYT), Harare MP163, Zimbabwe; ^d^College of Basic and Applied Sciences, Forest and Horticultural Crops Research Centre, University of Ghana, Legon Accra LG25, Ghana; ^e^International Institute of Tropical Agriculture (IITA), Council for Scientific and Industrial Research (CSIR) Campus, Legon Accra LG56, Ghana; ^f^International Institute of Tropical Agriculture (IITA), Dar es Salaam 34441, Tanzania; ^g^Department of Soil Science, Federal University of Technology Minna PMB65, Nigeria; ^h^Phoenix University Agwada, Nasarawa 1019, Nigeria; ^i^Crop Science Department, University of Zimbabwe, Zimbabwe/International Maize and Wheat Improvement Center (CIMMYT), Sustainable Agrifood Systems Program (SAS), Harare MP163, Zimbabwe; ^j^Department of Agronomy and Horticulture, University of Nebraska–Lincoln, Lincoln, NE 68583-0915; ^k^Earth Observation and Environmental Informatics, Wageningen Environmental Research, Wageningen University & Research, Wageningen 6700AA, The Netherlands; ^l^Département des Cultures Pluviales, Institut National de la Recherche Agronomique du Niger, Niamey BP 429, Niger; ^m^Foresight and Policy Modeling Unit, International Food Policy Research Institute (IFPRI), Washington, D.C. 20005; ^n^Department of Soil Science and Land Use Management, Makerere University, Kampala 7062, Uganda; ^o^Climate Adaptation, Sustainable Agriculture and Resilience Unit, AGRA, Nairobi 66773, Kenya; ^p^National Key Laboratory of Crop Genetic Improvement, Hubei Hongshan Laboratory, Ministry of Agriculture and Rural Affairs of the People’s Republic of China (MARA) Key Laboratory of Crop Ecophysiology and Farming System in the Middle Reaches of the Yangtze River, College of Plant Science and Technology, Huazhong Agricultural University, Wuhan 430070, China

**Keywords:** food availability, cereal demand, climate change, yield potential, crop area

## Abstract

Sub-Saharan Africa (SSA) has the world’s largest projected increase in demand for food. Increased dependence on imports makes SSA vulnerable to geopolitical and economic risks, while further expansion of agricultural land is environmentally harmful. Cereals, in particular, maize, millet, rice, sorghum, and wheat, take nearly 50% of the cropland and 43% of the calories and proteins consumed in the region. Demand is projected to double until 2050. Here, we assess recent developments in cereal self-sufficiency and provide outlooks until 2050 under different intensification, area expansion, and climate change scenarios. We use detailed data for ten countries. Cereal self-sufficiency increased between 2010 and 2020 from 84 to 92% despite the 29% population increase. The production increase was achieved by increased yields per hectare (44%), area expansion (34%), and a shift from millet to the higher yielding maize (22%). Outlooks for 2050 are less pessimistic than earlier assessments because of the larger 2020 baseline area, higher shares of maize and somewhat less steep projected population increase. Yet, to halt further area expansion, a drastic trend change in annual yield increase from the present 20 to 58 kg ha^−1^ y^−1^ is needed to achieve cereal self-sufficiency. While such yield increases have been achieved elsewhere and are feasible given the yield potentials in SSA, they require structural changes and substantial agronomic, socioeconomic, and political investments. We estimate that amounts of added nitrogen need to at least triple to achieve such yield improvements, but it is essential that this comes with improved context-specific agronomy.

## Significance

Some 54% of the world’s population increase until 2050 will occur in sub-Saharan Africa (SSA), while the region has the highest incidence of malnutrition. Its food demand is projected to double between 2020 and 2050. Previous assessments of SSA’s potential to achieve cereal self-sufficiency were pessimistic. Here, we show self-sufficiency increased between 2010 and 2020, despite a 29% increase in population, due to area expansion, changes in grown cereal crops and yield increases. The larger baseline area in 2020, a larger share of maize and lower projected population increase make achieving cereal self-sufficiency by 2050 more feasible. Yet, large yield gains are needed to avoid further import dependency and cropland expansion which is detrimental for biodiversity and greenhouse gas emissions.

Many studies in agricultural sciences seek their justification in ever-increasing food demand. The latest projections vary between 35 to 60% increase in food demand between 2020 and 2050 (1, 2). This increase expressed in the monetary value of food is driven by a projected 1.8 billion population increase, the strong relationship between income and the consumption of animal proteins (3), and the increasing demand for biomass from a more circular economy (4, 5). Some 54% of the increase in population by 2050 will live in sub-Saharan Africa (SSA) (6). And it is also this part of the world where the highest percentage of people cannot yet afford a healthy diet (7). Arguably, SSA must be the prime focus of increasing food availability, because the continent is not self-sufficient (just below 80%) today (8) and because productivity growth remains slow (9, 10).

Both the availability and prices of agricultural products in the world market are highly affected by global shocks. In recent years, multiple factors, such as climate conditions (11), the COVID−19 pandemic (12), and geopolitical conflicts (13) played a role in these market shocks. Consequently, national and regional governments and institutions (incl. the African Union) increased their attention to attaining a higher degree of self-sufficiency of agricultural products to safeguard national or regional security and lowering the influence of global market fluctuations on their food security (14, 15). Low- or middle-income countries, like those in SSA, face an even greater challenge due to their limited purchasing power in global food markets compared to high-income countries (16). In addition, some of the world’s major exporting countries have not shown a significant growth in agricultural production in recent years because of yield stagnation and sometimes cropland contraction (17–19). In the face of global market shocks and the rapidly increasing food demand in SSA, food security in the region, and consequently national security of its countries, is fragile (20). Also, investing in local food production strengthens rural economies through job creation in the food system (21, 22) and hence supports economic growth (14). All these arguments justify a strong focus on current and future food production capacity in SSA, more so since the region is the only one that is using cropland area expansion (sometimes also labeled as agricultural extensification) as the dominant pathway to increase production (9, 23). Given finite scope for further area expansion (24, 25) and the important trade-offs between area expansion and biodiversity conservation and greenhouse gas emissions, increasing yields on existing cropland with good agronomy is the less harmful pathway (26–28).

It is in this context that the strong focus on intra-African trade by many regional economic institutions such as the African Union and the African Development Bank must be seen, as reflected in e.g., Agenda 2063 and the African Continental Free Trade Area (29). We note that full self-sufficiency at the regional, let alone national level, is rarely an economically justified target. Yet, self-sufficiency and the degree to which this can be achieved on current crop acreages is an indicator of production challenges and opportunities at both the regional and national levels. It can help guide investment decisions and policy making to enhance the use of comparative advantages across the region and hence increase overall efficiency and resilience of Africa’s agrifood systems.

Cereals play a vital role in ensuring food security in SSA, as these account for ca. 49% of croplands and ca. 43% of both the caloric and protein intake (30). However, productivity of cereals and other crops is low in SSA, as indicated by the large difference between their potential and actual yields, i.e., the yield gap (9). The potential yield (Y_p_) is defined as the maximum yield of a locally adapted crop cultivar in the absence of abiotic (water and nutrients) and biotic (weeds, pests, and diseases) stresses. In the case of rainfed crops, the water-limited yield (rainfed) potential (Y_w_) is the benchmark as it is also determined by precipitation patterns and soil properties influencing the crop water availability (31). Actual cereal yields achieved by farmers in SSA are often only ca. 20 to 40% of Y_p_ or Y_w_ (32). Narrowing this gap is a viable strategy for achieving improved cereal self-sufficiency in SSA (9). Yet, improving cereal yields is challenging, as inherent soil fertility in SSA is low (33, 34) and enhancing soil fertility with adequate nutrient fertilization is essential (35, 36). Van Ittersum et al. (9) concluded that yield gap closure of cereals on existing cropland, using data up to the year 2010, would allow for cereal self-sufficiency by 2050, but only just, and it would require an unprecedented trend change and steep increase in cereal yields. Given the importance of the topic for the future of SSA and the world, and the potentially fast developments in terms of changes in population growth, cropland area, climate change (37, 38) and crop yields in some countries (39, 40), it is timely to reassess SSA’s outlook. In this paper, we assess this in a comprehensive manner by considering recent developments in terms of crop area and cropping pattern changes (defined as changes in area shares of the different cereals grown) as well as yield changes, and the effects of future climate change.

We use data for five cereals (maize, millet, rice, sorghum, and wheat) and ten SSA countries (Burkina Faso, Ghana, Mali, Niger, and Nigeria in West Africa, jointly labeled WA; Ethiopia, Kenya, Tanzania, Uganda, and Zambia in East and Southern Africa, jointly labeled ESA, and the ten countries together are hereafter labeled SSA). Together, the ten countries capture 54% of SSA’s population and 61% of SSA’s cereal area, hence the selected countries are relatively cropland abundant (30). Our objective was to assess i) developments in terms of cereal areas, cropping patterns, yields, and self-sufficiency in the period 2010 to 2020, and ii) prospects in terms of cereal self-sufficiency and associated crop nutrient requirements by the year 2050.

We define the national and regional cereal self-sufficiency as production as a percentage of demand, respectively, at national and regional levels. All five cereal yields were converted into maize equivalents, using their own standard moisture content and caloric content. Several detailed datasets collected for the ten countries were complemented with national data and literature review to estimate cereal demand and production (*Methods*). Cereal demand was calculated as the per capita consumption multiplied by the population (*SI Appendix*, *Supporting Section* 1). Cereal production was calculated as yield [source GYGA (32)] multiplied by area, in which area was a function of physical area [based on SPAM2010 and SPAM2020–(41, 42)] and cropping intensity [taken from GYGA (32)]. For the assessments for 2010 and 2020, actual cereal yields and acreages were used, while for the time horizon 2050, five different production scenarios (Table 1) were evaluated taking into account local yield potentials and effects of climate change, and the fact that in all scenarios yields cannot exceed 80% of the country-specific yield potential. The five scenarios were the following: i) Y_2020_, both yields and areas are the same as in 2020 despite projected changes in population and diet; this is an unlikely scenario but used for comparative purposes; ii) Y_trend_, yields will rise until 2050, following the same trends as from 2010 to 2020 and crop areas will remain the same as in 2020; iii) Y_trend_A_trend_, captures the combined effects of increasing cereal yields and expanding cereal areas based on current trends (i.e., between 2010 and 2020); iv) Y_ss_, required yield levels until complete self-sufficiency, while cereal areas remain the same as in 2020; v) Y_ss_A_trend_, required yield increases needed to achieve self-sufficiency, while cereal area expansion follows the 2010 to 2020 trend. Results of the 2050 scenarios are presented in the main text for the Shared Socio-economic Pathway 2 (SSP2) which represents a medium scenario in terms of sustainable development (43), while results for the relatively optimistic SSP1 and pessimistic SSP3 are presented in the SI. Actual crop nutrient use (nitrogen - N, phosphorus – P, and potassium - K) in 2020 was based on FAOSTAT (44), while so-called minimum crop nutrient requirements for yields and production in 2020 and 2050 were estimated according to Ten Berge et al. (36) assuming highly efficient nutrient use.

**Table 1.** Self-sufficiency scenarios for 2050 with different assumptions about yield gains and cereal area expansion

Scenario	Yield gain pathway	Area expansion pathway
Y_2020_	Yield in 2050 equals actual yield in 2020	Area in 2050 equals area in 2020
Y_trend_	Yield increases until 2050, capped at 80% of the yield potential, following the trend in actual yield between 2010 and 2020 Eq. **5**	Area in 2050 equals area in 2020
Y_trend_A_trend_	Yield increases until 2050, capped at 80% of the yield potential, following the trend in yield intensification Eq. **5** and cropping pattern effect Eq. **4** of the 2010 to 2020 period	Increases in cereal area (also of individual crops, which means cropping patterns may also change) based on the trend of the 2010 to 2020 period in each country. If the trend was negative for a given crop between 2010 and 2020, the area of 2050 was assumed the same as in 2020
Y_ss_	Yields increase until full self-sufficiency by 2050, but capped at 80% of yield potential	Area in 2050 equals area in 2020
Y_ss_A_trend_	Yields increase until full self-sufficiency by 2050, but capped at 80% of yield potential	Same as the scenario Y_trend_A_trend_

Cereal yields in the Ytrend, YtrendAtrend, Yss, and YssAtrend scenarios were capped at 80% of yield potential Eq. **6** if these surpassed this threshold. Estimations of yield potential in 2050 included effects of future climate change (including elevated atmospheric CO2 concentration).

## Results

### Cereal Self-Sufficiency Developments between 2010 and 2020.

**Fig. 1.**
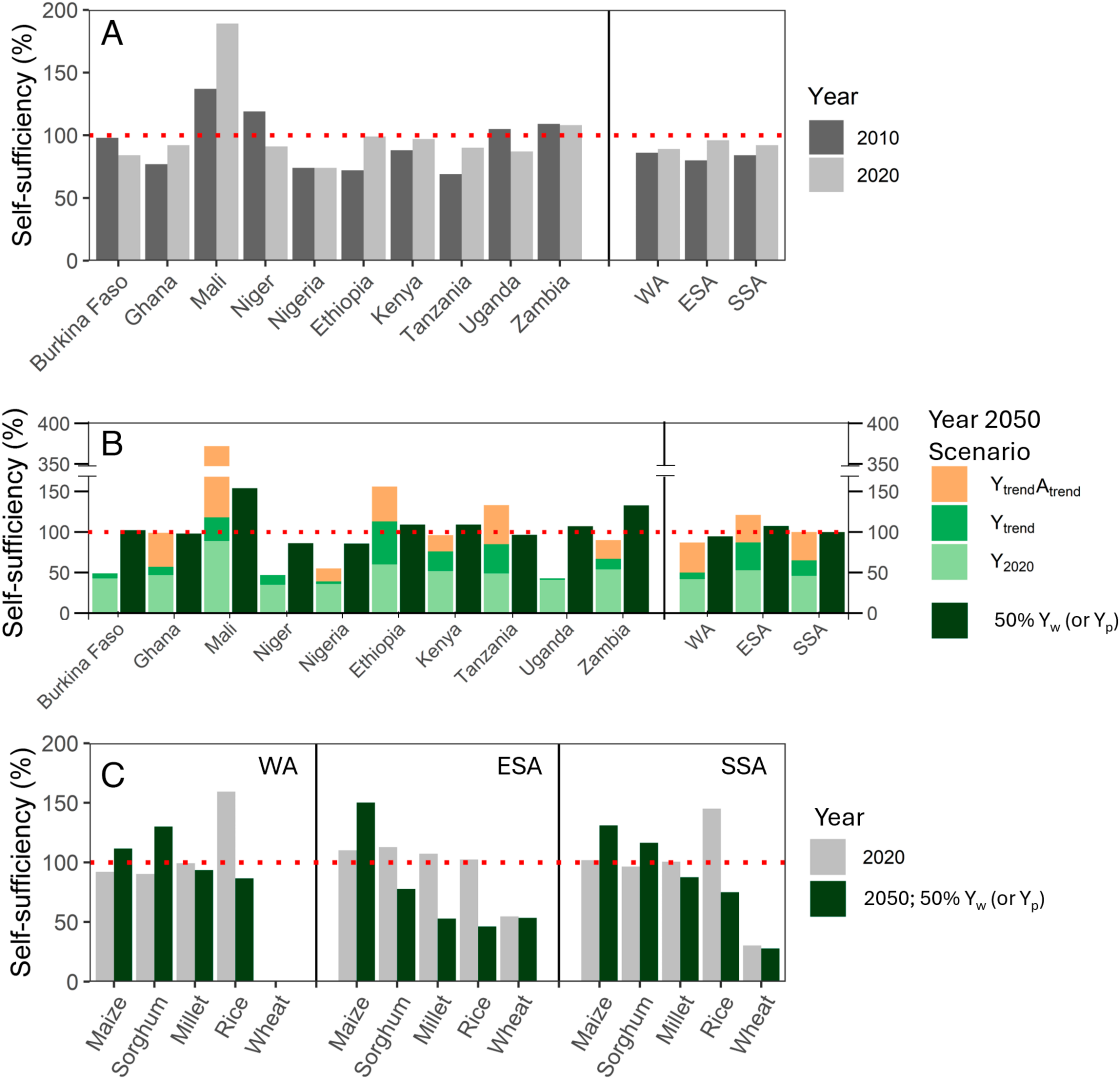
Current (2010 and 2020) and future (2050) cereal self-sufficiency in SSA based on different production scenarios. Panels (*A* and *B*) show cereal self-sufficiency for the five cereals, expressed in maize equivalents. For 2050, cereal demand was calculated based on SSP2. The three displayed scenarios (Y_2020_, Y_trend_, Y_trend_A_trend_) use historical or trend-based yields and crop areas; as a reference, self-sufficiency for cereal yields of 50% of yield potential (Y_w_ for maize, millet, rainfed rice, sorghum, and wheat, and Y_p_ for irrigated rice) on 2020 cereal acreages is also shown. Panel (*C*) shows self-sufficiency for each of the five cereals for 2020 and 2050 (50% of yield potential). The red dotted line represents a cereal self-sufficiency of 100%. Further information about scenarios is provided in Table 1. WA = the five countries in West Africa; ESA = the five countries in East and Southern Africa; SSA = the ten countries in sub-Saharan Africa.

**Fig. 2.**
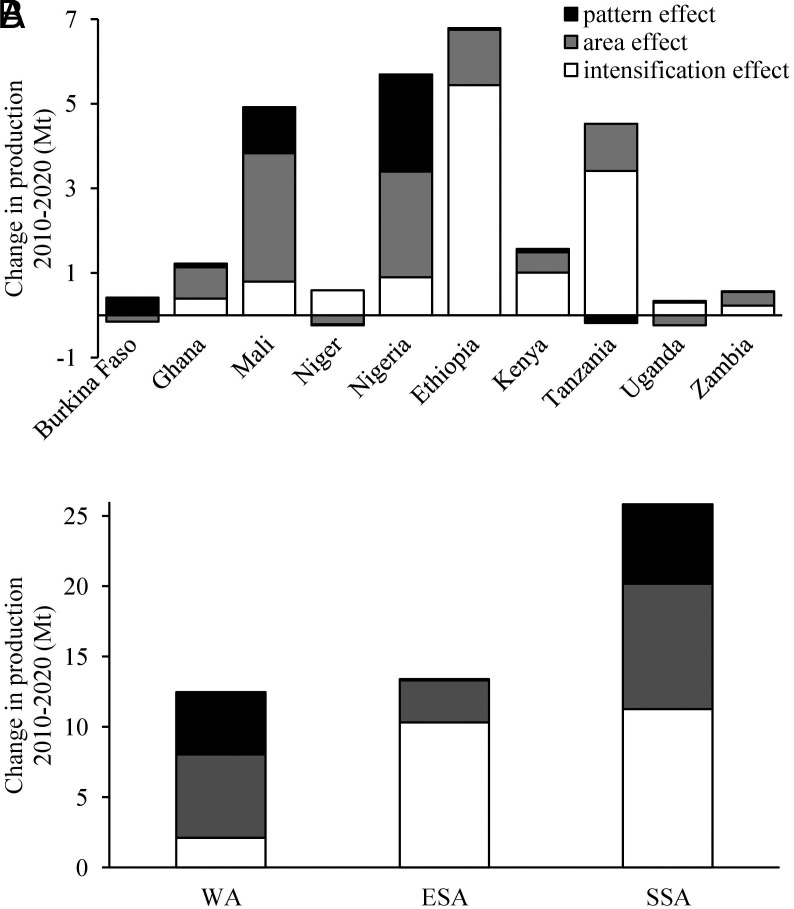
Attribution of cereal production changes between 2010 and 2020 to crop intensification, area expansion, and cropping pattern change. Data are displayed for each country separately (*A*) and the aggregated values for the five countries in West Africa (WA), five countries in East and Southern Africa (ESA), and the ten countries (SSA) (*B*).

Developments between 2010 and 2020 resulted in an increase of cereal self-sufficiency from 84 to 92% in SSA, with ESA showing a much stronger increase (from 80 to 96%) than WA (from 86 to 89%) (Fig. 1*A*). The higher cereal self-sufficiency in SSA was the result of the increase in cereal production (25.8 Mt) exceeding the increase in cereal demand (21.9 Mt) (*SI Appendix*, Table S1). While population growth rates were similar in WA and ESA (ca. 2.8% y^−1^, *SI Appendix*, Table S1), cereal production improved much faster in ESA (4.8% y^−1^) than in WA (3.3% y^−1^). In WA, Nigeria showed the most substantial increase in production, accounting for 46% of the region's total production growth between 2010 and 2020. In ESA, Ethiopia contributed over 50% to the total increase in production (Fig. 2). Evidently, the aggregated cereal self-sufficiency expressed in maize equivalents masks substantial differences between the five cereals (Fig. 1*C*), being lowest for wheat in ESA.

For the ten countries in SSA aggregated, the production increase was achieved by increased yields per hectare (44%), area expansion (34%), and a shift from millet to the much higher yielding maize (22%) (Fig. 2). However, the increase of cereal production in WA and ESA had distinct causes (Fig. 2). In WA, the prime driver was expansion of cereal area, i.e., 12% area increase, resulting in 5.9 Mt more production (accounting for 47% of the increased production). The second driver in WA was the change in cropping pattern, i.e., 4.4 Mt extra production (36% of the total increase) was attributed to an increased area share of maize at the expense of millet (*SI Appendix*, Table S2). The role of intensification (yield increase per ha) was relatively small in WA, i.e., 2.1 Mt (17% of the increase). In ESA, in contrast, intensification accounted for 77% of the increase in cereal production (10.3 Mt), area expansion for 22% (3 Mt), while changes in the cropping pattern were negligible (Fig. 2). Note, there were significant differences between countries within both regions (Fig. 1*A*). Niger and Uganda, for example, were more than self-sufficient in 2010, but their cereal self-sufficiency was well below 100% in 2020 (it decreased from 119 to 91% and from 105 to 87%, respectively). At the same time, Ethiopia and Mali showed large increases in cereal self-sufficiency, leading to full self-sufficiency in Ethiopia (from 72 to 99%) and much more than that in Mali (increase from 137 to 189%).

Despite increases in cereal production and self-sufficiency in the past decade, we note cereal production in the region is often affected by climate and other shocks that pose serious challenges to cereal self-sufficiency in specific years, with lasting effects beyond the specific year. When looking at the pattern and variability of cereal production progress in the ten countries over the 2000 to 2021 period, we can observe significant negative deviations from the trend in four out of 22 y due to extremely warm and dry conditions (deviations up to 12% from the trend line) (*SI Appendix*, Fig. S1). In the other 18 y, the annual cereal production deviated only ca. 2% from the trend line.

### Looking Ahead to 2050.

#### Cereal self-sufficiency scenarios.

SSA’s cereal self-sufficiency would decline from 92% in 2020 to 46% in 2050 if neither yields nor areas increase during that period (Y_2020_ scenario; Fig. 1*B*). By 2050, cereal demand in SSA is projected to be 98 Mt higher than in 2020, with 63 Mt in WA and 35 Mt in ESA. Both the lower population growth and lower increase in per capita demand in ESA than in WA explain the smaller increase in cereal demand in ESA (*SI Appendix*, Table S3). Differences between the three SSPs in total cereal demand were less than 10%, despite relatively large effects of the SSPs on population growth and per capita demand changes as these largely compensated each other (*SI Appendix*, Table S3).

**Fig. 3.**
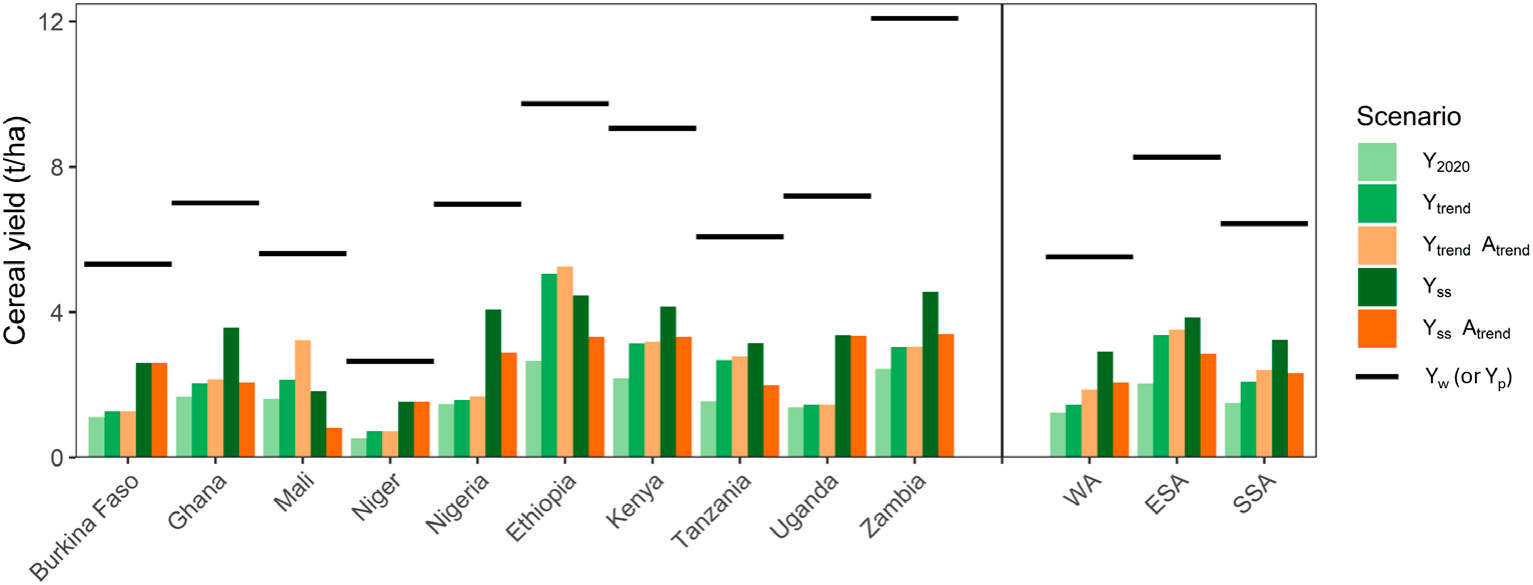
Cereal yields under different yield gain and area expansion scenarios in 2050 across SSA. The black horizontal lines indicate the yield potential (Y_w_ for rainfed crops and Y_p_ for irrigated rice) of the cereals expressed in maize equivalents (t ha^−1^). The first three scenarios (Y_2020_, Y_trend_, Y_trend_A_trend_) use historical or trend-based yields and crop areas, while the last two scenarios use yield targets needed to reach cereal self-sufficiency assuming current (year 2020) cereal areas (Y_ss_) or areas that increased following the recent historical trend (Y_ss_A_trend_). Further information about scenarios is provided in Table 1. WA = the five countries in West Africa; ESA = the five countries in East and Southern Africa; SSA = the ten countries in sub-Saharan Africa.

If yield trends of the period 2010 to 2020 continue until 2050 (Y_trend_ scenario), self-sufficiency of SSA could reach 65% in 2050 (Fig. 1*B*). In this scenario, ESA is expected to have a much higher cereal self-sufficiency (87%) than WA (50%) because cereal yields in WA have hardly increased in the 2010 to 2020 period (Fig. 3 and *SI Appendix*, Table S1). Note that Mali’s cereal self-sufficiency drops sharply in this scenario although still exceeding 100%, while Ethiopia is projected to achieve cereal self-sufficiency with the Y_trend_ scenario (Fig. 3).

Full cereal self-sufficiency can be reached in SSA if both yields and area continue to change according to the 2010 to 2020 trends (Y_trend_A_trend_ scenario) (Fig. 1*B*). This scenario assumes an (undesirable) area increase from 61.0 Mha in 2020 to 81.9 Mha in 2050, with increases of 14.3 Mha in WA and 6.6 Mha in ESA (*SI Appendix*, Table S3). The resulting cereal self-sufficiency for 2050 in WA is 87% and in ESA 121% (Fig. 1*B*). Despite the much higher projected area expansion in WA than in ESA, WA will not reach cereal self-sufficiency in this scenario. Here also, important differences between countries within both regions are notable. Mali, for example, increases cereal self-sufficiency due to a massive, hypothetical, area expansion from 6.3 Mha in the Y_trend_ scenario to 13.2 Mha in the Y_trend_A_trend_ scenario. Tanzania would achieve cereal self-sufficiency thanks to an assumed substantial area expansion from 6.4 to 9.6 Mha following the 2010 to 2020 trend (Fig. 1*B*).

The fourth scenario (Y_ss_) projects complete cereal self-sufficiency by 2050 due to higher yields on 2020 cereal areas, corresponding to about 50% of yield potential in most countries (Figs. 1 and 3). For SSA, this means a cereal yield increase from 1.5 t ha^−1^ in 2020 to 3.2 t ha^−1^ in 2050 (*SI Appendix*, Table S3), which equals an increase of 58 kg ha^−1^ y^−1^. The current yield increase with constant cropping pattern is 20 kg ha^−1^ y^−1^ (*SI Appendix*, Table S1). In WA, cereal yields must increase from 1.2 t ha^−1^ in 2020 to 2.9 t ha^−1^ in 2050 (*SI Appendix*, Table S3), which is 7.4 times more than the current yield trend in the region (i.e., 6.4 kg ha^−1^ y^−1^, *SI Appendix*, Table S1). For instance, Nigeria and Ghana would require large yield increases, 17.4 and 3.8 times more than the current yield trend, respectively. By comparison, cereal yields in ESA must increase from 2.0 t ha^−1^ in 2020 to 3.9 t ha^−1^ in 2050, being 1.2 times more than the current yield trend of 51 kg ha^−1^ y^−1^ (*SI Appendix*, Table S1). As noted above, Ethiopia would achieve self-sufficiency already with the current yield trend (Fig. 1*B*).

In the fifth scenario with higher yields and a continuation of area expansion (Y_ss_A_trend_ scenario), cereal yields should increase to 2.3 t ha^−1^ in 2050 in SSA, which is 28% less than in the fourth scenario, Y_ss_ (Fig. 3). In WA, the target cereal yield in this scenario is 2.1 t ha^−1^, and in ESA 2.9 t ha^−1^ (Fig. 3 and *SI Appendix*, Table S3).

We acknowledge that expressing self-sufficiency of the five cereals in maize equivalents hides large differences, including regional differences, in import dependency between the five commodities. With current cereal cropping patterns, prospects for self-sufficiency of maize and sorghum are substantially better than for rice and in particular wheat (Fig. 1*C*).

#### Climate change impacts and relative yields needed for cereal self-sufficiency.

The previous section showed the opportunities to achieve cereal self-sufficiency, considering projected effects of climate change on rainfed (plus irrigated rice) yield potentials of the five cereals. Yield potential was most negatively affected by climate change for rice, while yield potential of sorghum and in particular wheat were projected to be positively affected by climate change in key producing countries [*SI Appendix*, Table S4; (38)].The negative effect of climate change on aggregated cereal yield potential was generally within 10%, with negative exceptions for Ghana and Mali and a positive exception for Niger where rainfall is projected to increase (*SI Appendix*, Table S4). Consequently, to achieve a given absolute yield target in 2020 and 2050 requires a larger relative yield gap closure (i.e., actual yield expressed as a percentage of the yield potential) in 2050 than in 2020 for all countries except Niger. Yet, the relative yield gap closure required to achieve cereal self-sufficiency by 2050, without cropland expansion, is within the range of target yields for which yield response to fertilizers is assumed linear (*Methods*), i.e., 53% of Y_w_ in WA, 47% of Y_w_ in ESA, and 50% of Y_w_ in SSA, being all smaller than 60% of Y_w_ (Figs. 1 and 3).

#### Actual crop nutrient use and minimum requirements for future yields.

It is relevant to explain the significant difference in recent historical cereal yield improvement between WA and ESA. In 2020, the theoretical minimum N requirement to produce one ton of cereal was 25 kg N t^−1^ grain in WA, while in ESA it was 22 kg N t^−1^ grain. Although small, this difference can be explained by differences in cropping pattern, i.e., maize is dominant in ESA and has a higher nutrient use efficiency than millet and sorghum, which are the dominant cereals in WA (*SI Appendix*, Tables S2 and S5).

**Fig. 4.**
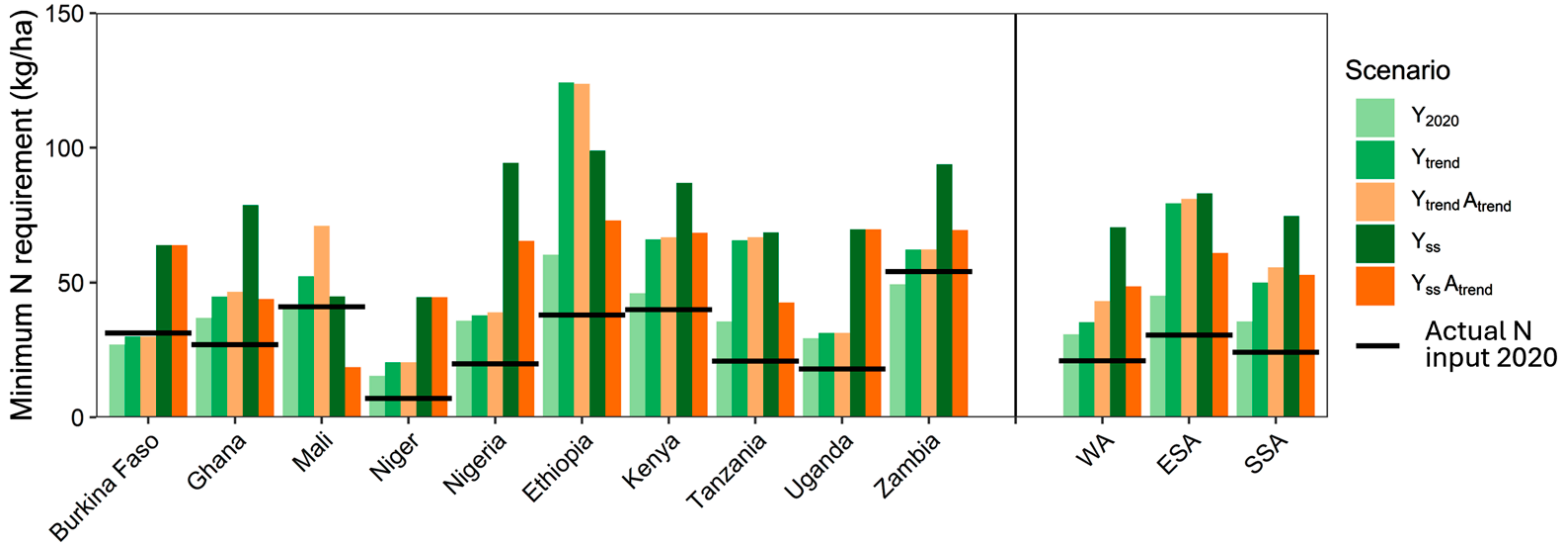
Minimum nitrogen (N) requirement under different yield gain and area expansion scenarios in 2050 across SSA. Solid lines represent the actual N input in 2020, the lightest green bar shows the minimum N requirement for the yield in 2020 (see *SI Appendix*, Fig. S1 for minimum P and K requirement and actual input). The first three scenarios (Y_2020_, Y_trend_, Y_trend_A_trend_) use historical or trend-based yields and cereal areas while the last two scenarios use yield targets needed to reach cereal self-sufficiency assuming current crop areas (Y_ss_) or areas that increased following the recent historical trend (Y_ss_A_trend_). Further information about scenarios is provided in Table 1. WA = the five countries in West Africa; ESA = the five countries in East and Southern Africa; SSA = the ten countries in sub-Saharan Africa.

Total actual N input (atmospheric deposition, manure, and mineral fertilizers) for cereals was estimated to be ca. 45% higher in ESA (31 kg ha^−1^) than in WA (21 kg ha^−1^) (Fig. 4). If we correct actual use of N in WA for the higher nutrient use efficiency in ESA due to the different cropping pattern, the amount of 21 kg N ha^−1^ used in WA would be equivalent to 18 kg N ha^−1^ (assuming the same cropping pattern as in ESA). Actual yields of cereals were 0.8 t ha^−1^ (66%) higher in ESA than in WA in 2020 (Fig. 3). Our analysis suggests that more than 70% of this yield difference between ESA and WA can be attributed to regional differences in total N input and use efficiency (the difference between the 31 kg N ha^−1^ usage in ESA and the 18 kg N ha^−1^ in WA corrected for cropping pattern, as a percentage of 18 kg N ha^−1^). When considering the N, P, and K inputs together, the conclusion is similar, because N input takes the largest share of the actual nutrient inputs (Fig. 4 and *SI Appendix*, Fig. S2).

In 2020, actual N input (deposition, manure, and mineral fertilizers) to cereals was 24 kg N ha^−1^ in SSA (31 kg N ha^−1^ in ESA and 21 kg N ha^−1^ in WA), while this amount of N was estimated to be enough to meet 68% of the crop N uptake in SSA (68% in both ESA and WA) (Fig. 4). The *minimum* N requirement for yield levels realizing cereal self-sufficiency in 2050 (Y_ss_ scenario) was estimated at 75 kg N ha^−1^ y^−1^ in SSA, 70 kg N ha^−1^ y^−1^ in WA, and 83 kg N ha^−1^ y^−1^ in ESA (Fig. 4).

A comparison between the Y_ss_ and Y_ss_A_trend_ scenarios revealed that the minimum N requirements per ha were higher in Y_ss_ (Fig. 4) since target yields in the latter scenario without area expansion were higher. However, when computing the total N requirement (amounts per ha times total area), the minimum N requirements were similar for both scenarios (4.5 Mt for Y_ss_ compared to 4.3 Mt for Y_ss_A_trend_) because almost all yield targets were below 60% of the yield potential, which implies a linear relationship between yield and minimum nutrient requirements (*Methods*). The actual use of P and K as a fraction of the minimum requirement of these nutrients for actual yields was much lower than that of N (*SI Appendix*, Fig. S2).

## Discussion

### Recent and Future Developments in Cereal Self-Sufficiency.

Our reassessment of SSA’s outlook shows a challenging, but less pessimistic perspective on future opportunities for attaining cereal self-sufficiency by 2050 on current cereal area than earlier studies. While van Ittersum et al. (9) concluded that attaining cereal self-sufficiency in SSA by 2050 on current cereal acreages would require an unprecedented trend change in yield progress until 80% of the (mostly rainfed) yield potential in just 40 y (2010 was the base year in the previous study), our updated analysis indicates that SSA can already attain this target on the 2020 cereal area with approximately 50% of the rainfed yield potential. The remarkable difference between these studies can be attributed to three factors: i) a cereal area increase of 13% between 2010 [baseline of (9)] and 2020 (baseline for the present study) in particular in WA; ii) a strong change in cropping pattern with the higher yielding maize, and to a lesser extent rice, replacing the much lower yielding millet also in WA (45), and iii) projections of demand by 2050 are lower than those in the previous study, particularly because of lower population increase projections (14% lower in the present study for SPP2). Moreover, since actual yields increased substantially in some countries in East Africa (Ethiopia and Tanzania), the new outlook translates into more feasible, though still drastic, yield trend changes needed to attain cereal self-sufficiency by 2050. Assuming no further increase in cereal areas nor changes in cropping pattern, cereal yields would have to increase by 56 kg ha^−1^ y^−1^ in WA and 60 kg ha^−1^ y^−1^ in ESA rather than 77 kg ha^−1^ y^−1^ and 105 kg ha^−1^ y^−1^, respectively, as estimated by (9). Our newly estimated required yield increase is comparable to those historically achieved in other rainfed cropping systems (46, 47). Yet, these yield increases deviate firmly from the present average 20 kg ha^−1^ y^−1^ increase (*SI Appendix*, Table S1) and yield stagnation observed in many African countries (10). Clearly, substantial efforts are still needed to move away from area expansion as a prime driver of production increase in SSA (48).

Climate change is likely to add to the challenge of increasing yield trends in future. The negative effect of climate change on aggregated cereal yield potential was generally within 10% (38, 49–51) (*SI Appendix*, Table S4), but we acknowledge that these estimations did not account for changes in extreme events and their frequency of occurrence. The lower rainfed yield potentials under climate change give less scope to increase yields and will make the needed trend changes more challenging to realize. Yet, two factors must be mentioned to nuance this. First, negative effects of climate change up to 2050 are generally such that the needed relative yield gap closures for cereal self-sufficiency are well below the exploitable yield gap, which is assumed 70 to 80% of the yield potential (31). Second, adaptations to climate change were not considered in our assessment, while various studies indicate considerable scope for adaptation through, e.g., cultivar choice (38, 50).

While this assessment is less pessimistic than the earlier one, we must flag several cautions. First, the less pessimistic outlook is largely due to recent area expansion and cropping pattern changes as well as somewhat lower population increase projections, rather than substantial and steady yield increases in most countries, particularly in WA. The steep area increases in Mali and Tanzania cannot continue due to lack of suitable land (22) and associated biodiversity loss and increased greenhouse gas emissions (24, 28). Second, the increase in maize area at the expense of millet is possible because new drought-tolerant maize cultivars with a shorter growing period are employed, which allow the crop escape late-season drought stress in the new areas of maize cultivation in WA (45). Although in conditions with mild and little water stress maize is superior to millet and sorghum in nutrient use efficiency, maize has lower nutrient use efficiency when subjected to severe water stress conditions (52), a prevailing environmental condition in WA. Also, the three cereals show differences in yield variation across years, with millet typically showing the highest variation due to confounding with the environments in which the crop is grown (*SI Appendix*, Table S6). Evidently, there may be trade-offs between these different cereals in terms of yield potential and yield stability under more extreme biophysical conditions. Third, we flag that an increased specialization in maize leads to a less varied human nutrition (53). Fourth, we expressed cereal self-sufficiency in maize equivalents, yet the trends and possibilities of achieving self-sufficiency differ per crop. Maize and sorghum self-sufficiency outlooks in SSA are relatively positive, while import dependency is likely to remain for rice and wheat in future (Fig. 1*C*); (15, 54, 55).

Finally, further challenges beyond the 2050 horizon can be foreseen. The population in entire SSA is projected to increase by another 1.2 billion between 2050 and 2100 (6).Negative impacts of climate change are also likely to become more severe beyond 2050 (38). The trend analysis of historical cereal production (past 22 y) revealed that annual production differed up to 12% from the trend (*SI Appendix*, Fig. S1), due to extreme warm and dry conditions. How this may change in future is highly uncertain. Increases in extreme events are not adequately represented in climate change studies, as General Circulation Models still struggle to accurately simulate future extreme events in Africa despite advancements (56, 57). In addition, crop growth models are generally not yet equipped to reliably simulate extreme weather conditions (58, 59). Finally, it is also not possible to estimate how yields may vary simultaneously across a range of countries in a particular future year, as climate change projections are not year specific.

Changes in crop areas and yields as reported in the 2020 data compared to the 2010 data may reflect both real changes and improved data on crop areas (and yields) that have become available in time. It is widely acknowledged that data availability and quality is constraining analyses for SSA (60–63). We used agronomically robust datasets and where needed compared different datasets to propose corrections. For instance, comparison of FAO and IMPACT data revealed a substantial uncertainty in per capita cereal consumption in Mali, Nigeria, and Ethiopia (*SI Appendix*, *Supporting Section* 1). Furthermore, cropland data for SSA (42) did not adequately capture double cropping systems which are common in ESA, and we corrected these by making use of the GYGA data (*SI Appendix*, *Supporting Section* 2). In summary, we made use of the strengths of different global datasets, complemented with national data and literature review to provide the best possible estimations for cereal self-sufficiency now and in the future. Better or full coverage of SSA of our analysis requires detailed weather and agronomic data for the other countries, in particular in Central Africa.

A next step from a policy perspective, which is beyond the scope of our study, is how to stimulate intra-African trade to make up for national and subregional deficiencies in cereals and to strive for cereal self-sufficiency at regional and subcontinental level instead of country level. This requires assessing where and under which conditions striving for regional self-sufficiency is economically sensible and acceptable from a geopolitical standpoint.

### Adequate Crop Nutrition and Good Agronomy to Narrow Yield Gaps.

While we acknowledge there are opportunities to increase irrigation in SSA with potentially substantial yield benefits (*SI Appendix*, Table S8), currently the share of irrigated cereals is less than 2% of the total cereal area (42). Also, many irrigation schemes in Africa are not operational (64), and irrigation will likely be prioritized to high value crops with assured markets. There is broad consensus that poor soil fertility and low fertilizer inputs are the prime agronomic constraints to crop productivity in the region (34, 36, 65, 66). This is obvious for N, but also for P and K, certainly for the long-term, as failure to replenish P and K removed from the soil will result in the depletion of these nutrients (35, 67, 68). The comparison of actual N inputs with the minimum nutrient requirements reveals that even for today’s yields, N inputs (24 kg N ha^−1^ on average in the ten countries) are not meeting the minimum requirements for those yields (36 kg N ha^−1^) (Fig. 4). Moreover, we estimated that the minimum N inputs would need to increase to 75 kg N ha^−1^ on average to reach the target yields required to achieve cereal self-sufficiency in 2050, corresponding to more than three times the current N inputs. These assessments of minimum nutrient requirements assume highly efficient nutrient management that can only be achieved with good agronomy and with favorable input:output price ratios at the farm level (66). Today, this is generally not the case, so real nutrient requirements will be higher than the minimum amounts estimated in our study.

There is a notable difference between actual N inputs in Ethiopia, Kenya, and Zambia, compared to the other countries, which is reflected in cereal yields (Figs. 3 and 4). Analysis of historical data supports the potential of increased nutrient use is often associated with subsidy programs targeting mineral fertilizer and other inputs (69). Ethiopia is a clear example, where there has been a change in the quantity and type of fertilizers in recent years (*SI Appendix*, Fig. S3) contributing to yield increases over time [83 kg ha^−1^ y^−1^ between 2010 and 2020, (39, 40). This suggests the feasibility of the required 56 to 60 kg ha^−1^ y^−1^ cereal yield increase which is required to attain self-sufficiency in SSA. Our analysis underpins the Nairobi declaration of the 2024 Africa Fertilizer and Soil Health Summit that emphasized the need to increase production, distribution, and actual use of fertilizers on the African continent.

Evidently, increased nutrient use will only be effective if accompanied by good contextual agronomy (34). A whole array of other agronomic factors must be dovetailed to increase crop productivity, including well-adapted high-yielding cultivars, good seed quality and quantity, tillage, timing of seeding and optimal planting density, timely and adequate weed, and pest and disease management (15, 54). In turn, this requires an enabling socioeconomic and political environment for the effective use of improved agronomy, particularly in a changing climate. While not trivial to realize, such enabling conditions are paramount to stop further cropland expansion and avoid import dependency in the region in the decades ahead.

## Methods

### Cereal Self-Sufficiency.

Cereal self-sufficiency is defined as the total cereal production as a percentage of the total cereal demand, all expressed in maize equivalents with standard moisture content. Standard moisture contents were used for all crops: 15.5% for maize, 14% for millet, rice, and sorghum, and 13.5% for wheat. All cereal yields were converted to maize equivalents (i.e., aggregated yield) using Eq. **1**:

[1] $$\mathrm{Yield}=\frac{\sum (Y_{c}*{\mathrm{Area}}_{c}*{\mathrm{EnCoef}}_{c})}{\sum ({\mathrm{Area}}_{c})},$$

[2] $${\mathrm{EnCoef}}_{c}=\frac{{\mathrm{EnCon}}_{c}}{{\mathrm{EnCon}}_{\mathrm{maize}}},$$

where Yield refers to the maize yield equivalent (t ha^−1^) of maize, millet, rice, sorghum, and wheat, based on their area shares; Y_c_ to the yield of crop c (t grain ha^−1^), and Area_c_ to the annual harvested area of crop c (ha). EnCoef_c_ is the energy coefficient of crop c (-); EnCon_c_ is the grain energy content of crop c (kcal kg^−1^) (*SI Appendix*, Table S7); and EnCon_maize_ is the maize grain energy content (kcal kg^−1^) (*SI Appendix*, Table S7). The procedures and data used to estimate cereal production and demand for the years 2010, 2020, and future scenarios for 2050 are explained below.

### Actual Yield and Area Data.

Actual yield (Ya) data were obtained from the Global Yield Gap Atlas (GYGA) (32) at the country level for five crops: rainfed maize, millet, rice, sorghum, and wheat, and rainfed and irrigated rice. The GYGA Ya data were derived from national statistics with the finest resolution available and mapped onto climate zones (70, 71). Thereafter they were aggregated to the national level based on weighted averages using harvested area. These national actual yields closely resemble actual yields as reported by FAOSTAT. We used the average data of two time periods covering the years 2001 to 2010 (labeled as Ya for 2010) and 2011 to 2020 (labeled as Ya for 2020). A coefficient of 0.70 was employed for the conversion of paddy rice to milled rice (72).

The area for each crop x country combination was extracted from (41) for the period 2001 to 2010 and from (42) for the period 2011 to 2020. As it is quite common for regions located near the equator to grow multiple crops on the same piece of land within a year (cropping intensity higher than one), we multiplied the area obtained from (41, 42) with the cropping intensity specific to each crop and country derived from GYGA, which is based on expert knowledge. This correction was necessary as (42) did not adequately capture cropping intensity in SSA (*SI Appendix*, *Supporting Section* 2). The harvested area corrected for cropping intensity showed good correspondence with the official statistics at the national level (*SI Appendix*, *Supporting Section* 2).

### Impacts of Area and Yield on Production Changes between 2010 and 2020.

Eq. **3** was used to quantify the effect of area change on cereal production change between 2010 and 2020 for each country:

[3] $$P_{\mathrm{area}}={\mathrm{Yield}}_{10}*({\mathrm{Area}}_{20}-{\mathrm{Area}}_{10}),$$

where P_area_ refers to cereal production change in 2020 compared to 2010 due to the area expansion (t maize equivalent); Yield_10_ to the aggregated cereal yield in 2010 calculated based on Eq. **1** (t maize equivalent per ha); Area_20_ to the total cereal area in 2020 (ha); and Area_10_ to the total cereal area in 2010 (ha).

The aggregated yield Eq. **1** can be affected by changes in the cropping area among the five cereal crops, and by their respective yields. Hereafter we call this the cropping pattern effect on yield Eq. **4**, or production when multiplied with the area.

[4] $${\mathrm{Yield}}_{\mathrm{pattern}}={\mathrm{Yield}}_{\left(Y20,\;Area20\right)}-{\mathrm{Yield}}_{\left(Y20,\;Area10\right)},$$

where Yield_pattern_ refers to the change between 2010 and 2020 in the aggregated yield of the cereals due to the cropping pattern change (t ha^−1^); Yield_(Y20, Area20)_ to the aggregated yield calculated by applying Eq. **1**, which incorporates both the yields (Y_20_) and area data (Area_20_) from the 2020 dataset (t ha^−1^); and Yield_(Y20, Area10)_ to the aggregated yield calculated by applying Eq. **1**, which incorporates the yields (Y_20_) from the 2020 dataset and area data (Area_10_) from the 2010 dataset (t ha^−1^).

Besides the changes in area and cropping pattern, the change in cereal production between 2010 and 2020 can also be attributed to yield increases resulting from better genetics, and crop management, hereafter referred to as crop yield intensification. Eq. **5** was used to quantify the effect of intensification on cereal yield:

[5] $${\mathrm{Yield}}_{\mathrm{intensification}}=(Yield_{\left(Y20,\;Area20\right)}-{\mathrm{Yield}}_{\left(Y10,\;Area10\right)})-{\mathrm{Yield}}_{\mathrm{pattern}},$$

where Yield_intensification_ refers to the change in the aggregated yield between 2010 and 2020 due to intensification (t ha^−1^); Yield_(Y20, Area20)_ to the aggregated yield calculated by applying Eq. **1**, which incorporates both the yield (Y_20_) and area shares of the five crops (Area_20_) from the 2020 dataset (t ha^−1^); Yield_(Y10, Area10)_ to the aggregated yield calculated by applying Eq. **1**, which incorporates both the yield (Y_10_) and area shares (Area_10_) of the five crops from the 2010 dataset (t ha^−1^); and Yield_pattern_ to the change in the aggregated yield between 2020 and 2010 due to cropping pattern change (t ha^−1^) derived from Eq. **4**

### Estimation of Yield Potentials With and Without Climate Change Impacts and Target Yields.

The potential yield of all five cereals under rainfed conditions (Y_w_), as well as the potential yield of rice under irrigated conditions (Y_p_) were obtained from GYGA for the period 2000 to 2019 (32). Hereafter, we refer to yield potential, which can refer to either Y_p_ or Y_w_ depending on whether crops are cultivated under irrigated or rainfed conditions. The GYGA approach estimates yield potential using local data (weather, soil, and crop management) from key production sites as input for crop growth simulation models [Hybrid-Maize2016 for maize; WOFOST 7.3 for millet, sorghum, and wheat; ORYZA2000v3 for rice (9, 38, 54, 55, 71)] in combination with a spatial framework for upscaling model outputs from reference weather stations to the national level (73). Critical to this approach is that model inputs and outputs are evaluated by local agronomic experts. We estimated the yield potential in 2050 using Eq. **6**:

[6] $$Y_{pot50(i,j)}=Y_{potcur(i,j)}*(1+CC_{\left(i,j\right)}),$$

where Y_pot50_ refers to the yield potential of crop i in country j under future climate conditions around 2050 (t ha^−1^) and Y_potcur_: yield potential of crop i in country j under the current climate (t ha^−1^). Yield potential data were extracted from the GYGA dataset. “CC” refers to the climate change impact on crop i in country j around the year 2050 relative to current climate conditions (*SI Appendix*, Table S4). The effects of climate change and CO_2_ increase (time horizon 2050 and assuming no adaptation) on yield potential were obtained from (38) for maize, millet, sorghum, and wheat under SSP370 and SSP585 scenarios, and from (49) for rice under RCP4.5 and RCP8.5 climate change scenarios.

We capped cereal yields at 80% of the yield potential, as the maximum yield achievable for farmers, in the calculation of the target yields for achieving cereal self-sufficiency in 2050 to account for diminishing returns to inputs which have both economic and environmental implications (31, 74, 75). To give an indication of the possible maximum effect of irrigation of rainfed cereals, we also estimated the irrigated yield potential (Y_p_) for rainfed maize, millet, sorghum, and wheat (*SI Appendix*, Table S8).

### Demand for Cereals.

For total cereal consumption (and hence cereal demand), we included direct human consumption, use as feed for livestock, and use as feedstock for biofuel. Per capita consumption was calculated as the total consumption divided by the population. Various sources (FAOSTAT, IMPACT) provide different values for the current per capita cereal consumption in the target countries, with significant discrepancies for some countries (*SI Appendix*, *Supporting Section* 1). Therefore, we adjusted the per capita cereal consumption data from FAOSTAT and IMPACT using values from national statistical reports (*SI Appendix*, *Supporting Section* 1). Due to lack of data, we assumed the same per capita consumption in 2010 and 2020. We further compared the cereal self-sufficiency based on our adjusted per capita demand estimations against those based on IMPACT2010 and IMPACT2020 (*SI Appendix, Supporting Section 1*) to investigate the sensitivity of self-sufficiency estimates to different per capita demand values.

To project the capita demand estimations to 2050, Eq. **7** was used, taking into account the potential influence of economic conditions and climate change on cereal consumption.

[7] $${\mathrm{Cap}}_{50j}={\mathrm{Cap}}_{\mathrm{curj}}*(1+CCP_{j}),$$

where Cap_50 j_ refers to the per capita consumption of cereals in 2050 (kg maize equivalent weight person^−1^ y^−1^) in country j; Cap_cur j_ to the per capita consumption of cereals in 2020 (kg maize equivalent weight person^−1^ y^−1^) in country j (*SI Appendix*, *Supporting Section* 1); and CCP_j_ to the relative change in per capita cereal consumption in country j between 2050 and 2020. This factor was calculated using the IMPACT dataset (76) for different shared socioeconomic pathways including SSP1, SSP2, and SSP3.

The population data per country in 2010 and 2020 were obtained from the IMPACT dataset (76, 77) (*SI Appendix*, Table S1). Data from the same shared socioeconomic pathways (SSP1, SSP2, SSP3) were used to determine the projected population per country in 2050, and the projected demand (*SI Appendix*, Table S3).

### Minimum and Actual Crop Nutrient Requirements.

We used the approach of Ten Berge et al. (36) to assess the long-term minimum crop nutrient input requirements. This approach assumes that annual application rates of macronutrients (nitrogen–N, phosphorus–P, potassium–K) should at least equal the total nutrient uptake in the aboveground crop biomass (grain and stover) at a given target yield. Note that the sources of these nutrients can be multiple, including organic and mineral fertilizers or atmospheric deposition. The internal use efficiency of nutrients (kg grain kg^−1^ nutrient uptake) of each crop was obtained from GYGA (*SI Appendix*, Table S5). For N, beyond target yields of ca. 60% of Y_w_ or Y_p_, it was assumed that N use efficiencies decrease, and minimum N input requirements increase more than linearly with yield levels due to accumulation in the crop (36). Actual nutrient input rates were taken from FAOSTAT (44), as used also in GYGA, and include nutrients from deposition, manure, and mineral fertilizers. We use the term minimum nutrient requirements because their calculation assumes high nutrient use efficiency, which is feasible, but requires excellent agronomic management and soil fertility (36). In practice, nutrient requirements will be higher if agronomy and soil fertility are not optimal, but the minimum nutrient requirements are considered an informative benchmark.

### Variability in Production Due to Historical Extreme Climate Factors.

Estimating large-scale temporal variation in cereal production under climate change is challenging. Projecting weather for specific years (for example the year 2050) is not possible and only probabilities of weather in specific years can be provided. The actual future weather will also be site-specific. It is thus difficult to make meaningful predictions of future variability in cereal yields and total cereal production for groups of countries. While we acknowledge that temporal variability in cereal yields and total production may increase in the future due to the increased occurrence of extreme events, we used temporal variation in (recent) historical total cereal production in SSA for the period 2000 to 2021 (30) as an indicator of weather variability in the future. To do this, we examined the aggregated cereal production trend over time using linear regression. First, we fitted a linear regression to production data from the 22 y available in the time series. Next, we excluded production data from years with a relative difference of more than −5% from the fitted regression, identifying these years as the ones with extreme weather impacts on cereal production (*SI Appendix*, Fig. S1). The omitted years were 2009, 2011, 2013, and 2021. Indeed, for these years severe climate impacts (drought and heat) on crop production were reported across large areas of SSA (78–80). Finally, we fitted a new linear regression to production data from the remaining 18 y. The relative difference between actual production reported by FAOSTAT for the four extreme years and the estimated production based on the adjusted trend was assumed to represent the maximum climate-driven declines in cereal production in the ten countries.

## Supplementary Material

Appendix 01 (PDF)

## Data Availability

RDS, CSV, XLXS data have been deposited in www.yieldgap.org and https://doi.org/10.17632/7s4frszjmz.2 (32, 81).
