# Supplementary material for: Prospects for cereal self-sufficiency in sub-Saharan Africa
Source: Proc Natl Acad Sci U S A. 2025 Jun 9;122(24):e2423669122. doi: 10.1073/pnas.2423669122 (PMC12184333; doi:10.1073/pnas.2423669122)
Supplement: Supplementary file 1 — Appendix 01 (PDF) [file pnas.2423669122.sapp.pdf]

PNAS

Supporting Information for
Prospects for cereal self-sufficiency in sub-Saharan Africa

Martin K. van Ittersum, Seyyedmajid Alimagham, João Vasco Silva, Samuel Adjei-Nsiah, Frederick P. Baijukya, Abdullahi Bala, Regis Chikowo, Patricio Grassini, Hugo L.E. de Groot, Aphrodis Nshizirungu, Abdelkader Mahamane Soulé, Timothy B. Sulser, Godfrey Taulya, Fatima Amor Tenorio, Kindie Tesfaye, Shen Yuan, Marloes P. van Loon

Martin K. van Ittersum
Email: martin.vanittersum@wur.nl

This PDF file includes:

Supporting Section 1 - Per capita demand estimation
Tables S1-1 to S1-2
Figure S1-1
Supporting Section 2 - Cropping intensity in FAOSTAT and SPAM
Figures S2-1 to S2-2
Table S2-1
Supporting Tables S1 to S8
Supporting Figures S1 to S3
SI References

Table of Contents

SI Section 1 - Demand 2020 estimation	3
SI Section 2 - Cropping intensity in FAOSTAT and SPAM	6
Supporting Tables	8
Table S1. Cultivated cereal area, aggregated actual cereal yields, annual yield increase, cereal production, population, per capita demand, and total demand for the years 2010 and 2020	8
Table S2. Share of harvested area per cereal crop in West and East and Southern Africa in 2010 and 2020	9
Table S3. Area, cereal yields, population, per capita and total cereal demand for different scenarios for the year 2050	10
Table S4. Projected relative impact of climate change on potential cereal yields in each country around 2050 compared to the 2020 potential yields	11
Table S5. Internal or physiological nutrient use efficiency of N, P, K for cereals	12
Table S6. Potential yields of each crop under current and future climate conditions	13
Table S7. Energy content of each cereal	14
Table S8. Percentage increase in yield potential under irrigated conditions compared to rainfed conditions	14
Supporting Figures	15
Figure S1. Trend in total annual cereal production	15
Figure S2. A) Minimum phosphorus (P) and B) potassium (K) requirements for the different countries and regions under different scenarios in 2050	16
Figure S3. Total amount and types of fertilizers used in Ethiopia from 2010 to 2022	17
SI References	18

SI Section 1 - Demand 2020 estimation

This section explains how the 2020 per capita consumption of cereals was estimated based on the best available data. The 2020 values are combined with the projected changes in demand between 2020 and 2050 derived from the IMPACT model [1]. While it is common practice to stick to one data source, the per capita consumption of cereals (i.e., maize, millet, rice, sorghum and wheat) shows a large difference between different sources for Mali, Ethiopia, and Nigeria and on top of that the consumption for Mali seems to be extremely high (Table S1-1). Since these three countries make up 57% of the total population in the ten countries (Table S1-1), it is critical to examine these differences and use an appropriate estimate for these countries.

Table S1-1. Per capita consumption of maize, millet, sorghum, rice, and wheat in the ten selected African countries (kg maize equivalents fresh weight person^{-1} yr^{-1}) for the year 2020.

Country	FAOSTAT*	IMPACT2020**	IMPACT2010***	CV (%)	**Proposed values******
Burkina Faso	246	244	260	3.5	**250**
Ghana	130	151	144	7.5	**142**
Mali	335	390	212	29.2	***264***
Niger	267	289	260	5.6	**272**
Nigeria	141	142	206	22.9	***175***
Ethiopia	190	218	131	24.8	***172***
Kenya	152	147	134	6.3	**144**
Uganda	97	84	106	11.9	**102**
Tanzania	139	144	159	7.0	***187***
Zambia	168	179	162	5.1	**170**

* average value for the time period from 2010 to 2021
** simulations using CMIP6 data [1]
*** simulations using CMIP5 data [2]
**** for Burkina Faso, Ghana, Niger, Kenya, Uganda, and Zambia we took the average of the three different sources in this table; for Mali, Nigeria, Ethiopia and Tanzania the proposed value was based on the regression of Fig. S1-1 and the contribution of cereals to the annual caloric intake of these countries from Table S1-2.

The contribution of the cereals to total caloric intake in each country was verified with national data from literature (Table S1-2). Following that, a linear regression model was derived (Fig. S1-1) with the per capita consumption values as dependent variable (last column of Table S1-1) and the contribution of cereals to the total annual caloric intake as independent variable (Table S1-2). Data for Mali, Ethiopia, and Nigeria were omitted due to the large differences in the per capita values between different sources (Table S1-1). We also omitted data of Tanzania for this regression, as the sub-national observations for this country showed significant differences compared with the data in Table SD1 [3]. As a result, only data from Burkina Faso, Ghana, Niger, Kenya, Uganda, and Zambia were used for the linear regression model (Fig. S1-1).

The slope of the regression between per capita cereal consumption and the contribution of the five cereals to the total annual caloric intake (Fig. S1-1) provides the required amount of maize equivalents per percent that the five cereals are contributing to the total annual caloric intake, being 2.97 kg person^{-1} year^{-1} (Fig. S1-1). Multiplying this value with the energy content of a maize equivalent (3500 kcal per maize equivalent) provides the required calories per percent that the five cereals are contributing to the total annual caloric intake (2.97 * 3500 = 10,395 kcal person^{-1} yr^{-1}). This is equivalent to a daily value of 28.47 kcal person^{-1} d^{-1} Assuming a daily caloric intake of 2250 kcal person^{-1} d^{-1}, one percent of this value would be 22.5 kcal person^{-1} d^{-1}; this can be considered a net value. The ratio between gross and net calories (28.48 / 22.5 = 1.27) suggests that 27% of the total cereal demand can be attributed to loss, waste and feed, a value which is rather consistent with data for SSA [4].

Following the application of the regression formula outlined in Fig. S1-1 and considering the caloric intake percentages derived from the five cereals according to the literature (Table S1-2), the per capita cereal consumption in Mali, Nigeria, Ethiopia and Tanzania was re-estimated (Table S1-1 last column). For the other countries, the average value of the three sources in Table S1-1 was used.

We compared the cereal self-sufficiency based on our adjusted per capita demand estimations with those based on IMPACT2010 [2] and IMPACT2020 [1] to investigate the sensitivity of our self-sufficiency estimates to different per capita demand estimations (Fig. S1-2). This revealed that effects of the different per capita demand estimations are relatively small for results aggregated to the level of WA, ESA, and in particular SSA. However, for individual countries differences can be large because of relatively large country-specific corrections (i.e., for Mali, Nigeria, Ethiopia, and Tanzania).

Table S1-2. The contribution of maize, millet, rice, sorghum, and wheat to the annual caloric intake in each country based on literature.

Country	Caloric intake from the five cereals (%)	Reference
Burkina Faso	74	[5]
Ghana	29	[6]
Mali	76	[7]
Niger	74	Assumed the same as in Burkina Faso
Nigeria	46	[8]
Ethiopia	45	[9]
Kenya	34	[10]
Uganda	26	[11]
Tanzania	50	[12]
Zambia	50	Assumed the same as in Tanzania

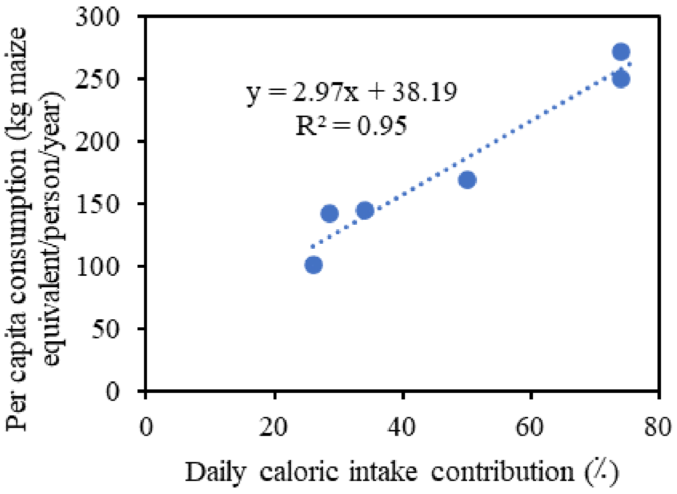

Figure S1-1. Linear regression between the per capita cereal consumption (last column of Table SD1) and the contribution of cereals to the annual caloric intake as derived from country-specific literature (Table SD2) for Burkina Faso, Ghana, Niger, Kenya, Uganda, and Zambia.

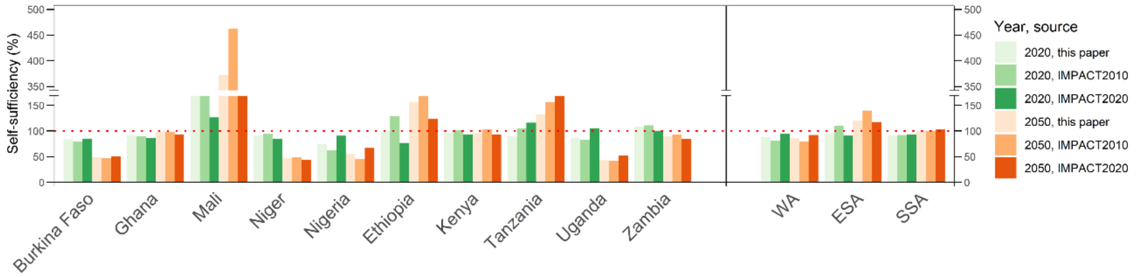

Figure S1-2. Cereal self-sufficiency for the year 2020 with current yields and for 2050 attained with cereal yields equal to 50% of Y_w (or Y_p for irrigated rice) for three different estimations of the per capita cereal demand: based on our approach (see above), IMPACT2010, and IMPACT2020 (Table S1-1).

SI Section 2 - Cropping intensity in FAOSTAT and SPAM

FAOSTAT's actual yield data show fluctuations which are hard to explain. An example of this is presented in Fig. S2-1, which demonstrates the unrealistic fluctuation in maize production in Uganda. A similar problem was found for millet in Ghana, maize in Ethiopia and Uganda, rice in Ethiopia, Kenya, and Uganda, sorghum in Ghana, and wheat in Tanzania. By comparing, for example, maize data for Uganda in 2018 and 2019 from national statistics (Table S2-1) with those from FAOTAT (Fig. S2-1), we can illustrate the plausible reason for the fluctuations in Fig. S2-1. The yields in 2018 and 2019 from FAOSTAT did not match the data in the national statistical reports. In FAOSTAT, since 2008 the yield for Uganda appears to represent the total of the yields from the first and second maize season, rather than an average of these two seasons. Moreover, the area represents the physical area rather than the harvested area (Fig. S2-1 and Table S2-1). Nonetheless, FAOSTAT's documentation states that the area for crop production is the harvested area, not the physical area [13]. It seems thus likely that the unusual fluctuation of the maize yield data for Uganda in FAOSTAT (Fig. S2-1) stems from the inconsistency in reported maize yield and area for countries with double cropping.

SPAM2020 [14] estimates Uganda's harvested maize area at 1.2 million hectares (Mha) and physical maize area at 0.7 Mha. However, national data indicates that these estimates represent about half of the actual harvested and physical maize area in the country (Table S2-1). While SPAM2020 accounts for a double cropping system in Uganda, it fails to accurately capture the total harvested and physical area. A key source of this discrepancy lies in the FAOSTAT database, which is a primary input for developing the SPAM2020 crop mask. In Uganda's case, FAOSTAT reports physical area instead of harvested area, introducing significant errors into the SPAM2020 estimates.

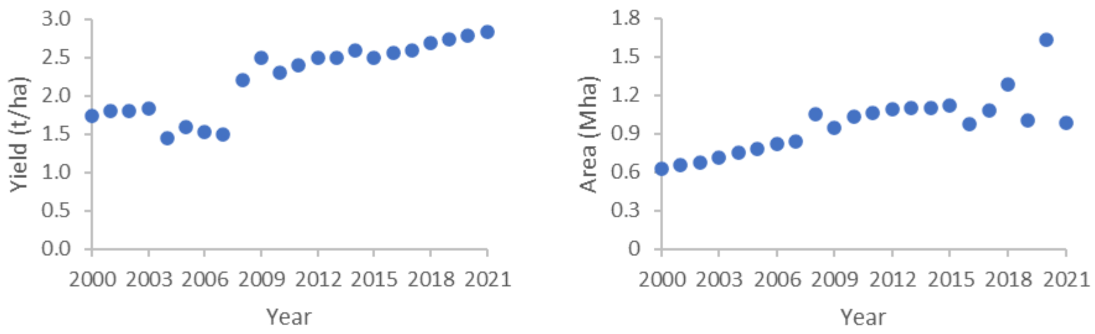

Fig. S2-1. Trends of maize yield and harvested area in Uganda derived from FAOSTAT [15].

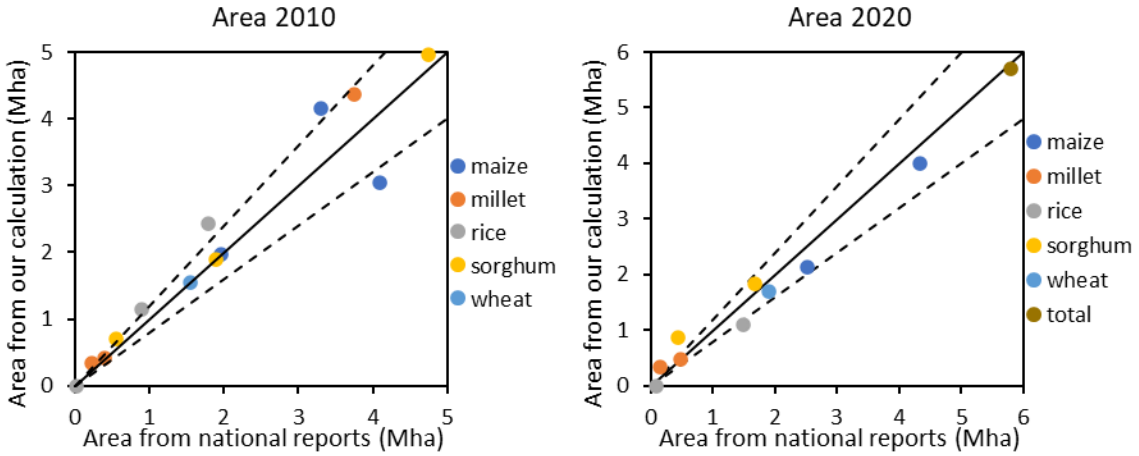

Fig. S2-2. Comparison of the area estimation calculated using our approach (see text for explanation) with the reported national annual data for various cereals in 2010 (average 2001-2010) and 2020 (average 2011-2020). The solid line represents a 1:1 relationship, and the dashed lines indicate a 20% deviation from the 1:1 line. Each point presents a country-crop combination; countries include those for which we had access to national data, i.e., Ethiopia, Mali, Nigeria, Tanzania, and Uganda.

Table S2-1. The data for maize area and yield in the first and second cropping season in Uganda [16, 17].

Year	First season		Second season		Total	
	Harvested Area (Mha)	Yield (t ha^{-1})	Harvested area (Mha)	Yield (t ha^{-1})	Harvested area (Mha)	Yield (t ha^{-1})
2018	1.5	1.4	0.8	1.7	2.3	1.5
2019	1.0	1.5	0.8	1.7	1.8	1.6
mean	1.2	1.5	0.8	1.7	2.0	1.5

Supporting Tables

Table S1. Cultivated cereal area, aggregated actual cereal yields, annual yield increase, cereal production, population, per capita demand, and total demand (all presented in fresh weight and in maize equivalents) for the years 2010 and 2020. WA: West Africa; ESA: East and Southern Africa; SSA: sub-Saharan Africa. Data sources, see Methods.

Location	Area (Mha)		Yield (t ha^{-1})		Annual yield increase (2010-2020) (kg ha^{-1} yr^{-1})			Production (Mt)		Population (millions)		Per capita demand (kg $person^{-1}$ yr^{-1})	Demand (Mt)	
	2010	2020	2010	2020	Intensification	Cropping pattern	Total	2010	2020	2010	2020		2010	2020
Burkina Faso	4.2	4.1	1.00	1.11	0.0	10.2	10.2	4.3	4.5	16	22	250	4.1	5.4
Ghana	1.8	2.4	1.47	1.67	16.7	3.4	20.2	2.7	3.9	24	30	142	3.5	4.3
Mali	4.0	6.3	1.31	1.62	12.7	17.4	30.2	5.2	10.1	15	21	264	4.1	5.4
Niger	10.6	10.1	0.48	0.53	5.8	0.0	5.8	5.0	5.4	16	22	272	4.2	5.9
Nigeria	15.9	17.8	1.28	1.46	5.0	12.9	17.9	20.4	26.1	158	203	175	27.7	35.2
Ethiopia	5.8	6.5	1.84	2.67	83.3	0.7	84.1	10.6	17.4	83	104	172	14.2	17.8
Kenya	3.0	3.3	1.83	2.17	30.9	2.5	33.4	5.5	7.1	41	52	144	5.8	7.4
Tanzania	5.3	6.4	1.06	1.55	50.8	0.0	50.8	5.5	9.8	45	59	187	8.4	10.9
Uganda	3.1	2.9	1.37	1.38	10.4	1.3	11.7	3.9	4.0	33	46	102	3.4	4.7
Zambia	1.1	1.3	2.22	2.44	17.6	1.2	18.8	2.5	3.1	13	17	170	2.2	2.9
WA	36.5	40.7	1.03	1.23	6.5	13.6	20.1	37.6	50.1	230	298	190	43.5	56.4
ESA	18.3	20.3	1.55	2.04	50.8	0.4	51.2	28.1	41.5	215	277	158	34.1	43.4
SSA (10 countries)	54.8	61.0	1.20	1.50	20.1	10.1	30.2	65.7	91.5	445	575	175	77.6	99.5
SSA total*	82.8**	95.8**	1.32	1.58	22.0	2.8	24.8	108.8	151.1	839	1019	178	149.1	181.3

* For the entire SSA, the data are from FAOSTAT [15] except for the per capita demand, which is from IMPACT [1] and our corrections for Mali, Nigeria, Ethiopia and Tanzania. In this case, area and yield for 2010 refers to the average of 2009, 2010, and 2011, and area and yield of 2020 to the average of the years 2019, 2020, and 2021.

** The five cereals accounted for 54% of all croplands in SSA in 2010 and 49% in 2020.

Table S2. Share of harvested area (%) per cereal crop in West and East and Southern Africa in 2010 and 2020 (based on SPAM2010 and SPAM2020).

	West Africa		East and Southern Africa	
Crop	2010	2020	2010	2020
Rainfed maize	18	24	58	63
Rainfed millet	40	29	7	5
Rainfed rice	8	12	7	5
Irrigated rice	2	3	0	1
Rainfed sorghum	32	32	19	16
Rainfed wheat	0	0	9	10

Table S3. Area, cereal yields, population, per capita and total cereal demand for different scenarios for the year 2050 (see Table 1 for explanation of scenarios). Data are own calculations derived from www.yieldgap.org (area trends and all yield data) or derived from SSP scenarios and the IMPACT model (population, per capita demand and total demand). WA = West Africa; ESA = East and Southern Africa, SSA = sub-Saharan Africa.

Location	Area (Mha)		Yield (t ha^{-1})									Population (millions)			Per capita demand (kg $person^{-1}$ yr^{-1})			Demand (Mt)		
	No area change	Area trend	Y_{2020}	Y_{trend}	$Y_{trend}A_{trend}$	Y_{ss}			$Y_{ss}A_{trend}$			SSP1	SSP2	SSP3	SSP1	SSP2	SSP3	SSP1	SSP2	SSP3
						SSP1	SSP2	SSP3	SSP1	SSP2	SSP3									
Burkina Faso	4.1	4.1	1.11	1.28	1.28	2.41	2.60	2.86	2.35	2.54	2.80	32.5	38.6	47.1	302	274	248	10	11	12
Ghana	2.4	3.9	1.67	2.04	2.15	3.51	3.57	3.65	2.03	2.06	2.11	39.9	46.4	54.5	207	181	157	8	8	9
Mali	6.3	13.2	1.62	2.15	3.23	1.77	1.82	1.88	0.80	0.82	0.85	30.4	35.9	42.0	364	318	281	11	11	12
Niger	10.1	10.1	0.53	0.72	0.72	1.35	1.53	1.71	1.35	1.54	1.72	38.7	50.9	63.8	353	305	272	14	16	17
Nigeria	17.8	23.7	1.46	1.59	1.67	3.89	4.07	4.34	2.77	2.89	3.09	328.6	371.7	433.7	212	195	179	70	73	78
Ethiopia	6.5	8.7	2.67	5.07	5.26	4.22	4.46	4.85	3.14	3.32	3.61	139.5	158.8	184.9	198	183	171	28	29	32
Kenya	3.3	4.0	2.17	3.14	3.19	4.29	4.15	4.36	3.43	3.32	3.48	70.3	78.1	95.9	198	173	148	14	14	14
Tanzania	6.4	9.6	1.55	2.68	2.78	2.89	3.15	3.42	1.82	1.99	2.16	85.0	102.3	120.7	216	196	180	18	20	22
Uganda	2.9	2.9	1.38	1.45	1.45	3.05	3.36	3.71	3.04	3.35	3.69	76.4	93.3	113.3	117	105	96	9	10	11
Zambia	1.3	1.7	2.44	3.03	3.05	4.41	4.55	4.94	3.29	3.40	3.68	25.5	30.1	37.9	221	193	166	6	6	6
WA	40.7	54.9	1.23	1.45	1.87	2.76	2.92	3.12	1.95	2.06	2.21	470.1	543.5	641.0	239	218	198	112	119	127
ESA	20.3	27.0	2.04	3.37	3.51	3.66	3.85	4.16	2.71	2.85	3.09	396.7	462.5	552.8	188	169	153	74	78	85
SSA	61.0	81.9	1.50	2.09	2.41	3.06	3.23	3.47	2.20	2.32	2.50	866.8	1006.0	1193.8	215	196	177	187	197	212

Table S4. Projected relative impact of climate change on potential cereal yields in each country around 2050 (average of 2040-2059) compared to the 2020 potential yields (average of 2000-2019) [18, 19]. 'Cereals aggregated' accounted for 2020 crop areas. Empty cells indicate that the cereal does not feature in that country or has a minor (<1% of total cropland area) acreage.

Country	Maize Rainfed	Millet Rainfed	Sorghum Rainfed	Wheat Rainfed	Rice Irrigated	Rice Rainfed	Cereals aggregated
Burkina Faso	-0.12	0.05	0.10	*	-0.19	-0.27	0.00
Ghana	-0.16	-0.24	-0.09		-0.19	-0.27	-0.17
Mali	-0.17	-0.11	0.03		-0.29	-0.11	-0.12
Niger		0.34	0.17		-0.25		0.28
Nigeria	-0.09	-0.03	0.14		-0.27	-0.25	-0.04
Ethiopia	-0.07	-0.33	-0.16	0.14		-0.16	-0.05
Kenya	-0.08	-0.08	0.00	0.11	-0.32		-0.07
Tanzania	-0.06	-0.21	-0.07	-0.01	-0.34	-0.19	-0.08
Uganda	0.01	-0.32	-0.23			-0.42	-0.05
Zambia	-0.08	-0.21				-0.47	-0.08

*empty cells indicate absence of crops or areas < 10,000 ha

Table S5. Internal or physiological nutrient use efficiency (kg grain kg^{-1} nutrient uptake) of N, P, K for cereals [20, 21].

Crop	Maximum accumulation			Medium dilution			Maximum dilution		
	N	P	K	N	P	K	N	P	K
Maize	35	208	31	50	416	78	64	625	125
Millet	15	74	14	32	196	53	48	317	91
Rice	21	140	23	50	341	55	79	542	87
Sorghum	19	115	21	42	230	31	64	345	41
Wheat	24	98	16	45	232	54	65	365	91

Table S6. Potential yields of each crop under current (2020; average of 2000-2019; data from Global Yield Gap Atlas [25]) and future (2050; average of 2040-2059) climate conditions (average of SSP370 and SSP585 for maize, millet, sorghum and wheat; and average of RCP4.5 and RCP8.5 for rice – see Table S4), with coefficients of variation (%) shown in parentheses. Yields are presented in fresh weight (t ha^{-1}), with rice yields based on paddy rice. WA = West Africa; ESA = East and Southern Africa; SSA = sub-Saharan Africa.

		Rainfed					Irrigated
Country	Time	Maize	Millet	Sorghum	Wheat	Rice	Rice
Burkina Faso	Current	7.8 (36.7)	3.3 (39)	5.3 (33)	*	6 (21.6)	8.9 (5.2)
Burkina Faso	Future	6.9 (41.2)	3.4 (36.4)	5.8 (27.8)		4.9 (17)	6.5 (9.9)
Ghana	Current	9.9 (20.8)	3.8 (18.4)	7.1 (16.1)		8.8 (11.2)	8.1 (3.9)
Ghana	Future	8.3 (24.4)	2.9 (25.1)	6.5 (16.2)		7.2 (16)	5.9 (7.1)
Mali	Current	10.7 (42.1)	3.6 (45.6)	6.7 (25)		6.4 (22.2)	9.1 (6.6)
Mali	Future	8.8 (48.5)	3.2 (54.5)	6.9 (20.7)		4.5 (19.3)	8.1 (13.6)
Niger	Current		2 (105.2)	2.2 (70.2)			9.3 (2.4)
Niger	Future		2.7 (86.2)	2.5 (63)			9.3 (11.6)
Nigeria	Current	11 (14.9)	2.7 (24.6)	6.7 (24.1)		7.6 (7.7)	8.5 (6)
Nigeria	Future	10 (14.5)	2.7 (24.4)	7.6 (19.9)		5.6 (6.6)	6.4 (12.1)
Ethiopia	Current	15.3 (11.4)	5.5 (13.9)	7 (15.1)	8.5 (19.9)	6.9 (19.3)	
Ethiopia	Future	14.3 (11.7)	3.7 (22.3)	5.8 (19.4)	9.7 (16.7)	6.9 (33.7)	
Kenya	Current	10.5 (29)	5.4 (20.7)	5.8 (19)	7 (41.1)		10.5 (1.6)
Kenya	Future	9.7 (24.9)	4.9 (20.8)	5.8 (15.7)	7.8 (37.3)		10.5 (4.8)
Tanzania	Current	8.1 (24.1)	2.3 (51.5)	3.1 (57.5)	3.8 (73.4)	8 (31.1)	10.8 (4.2)
Tanzania	Future	7.6 (29.6)	1.8 (47.7)	2.9 (51)	3.8 (74.7)	5.3 (22.6)	8.7 (5.2)
Uganda	Current	8.1 (36.4)	2.4 (25.5)	4.2 (22.7)		6.4 (32.5)	
Uganda	Future	8.2 (28.7)	1.6 (21.2)	3.3 (18.6)		6.4 (26.8)	
Zambia	Current	13.6 (12.7)	6.6 (10.1)			9.1 (25)	
Zambia	Future	12.5 (15.7)	5.2 (16.7)			9.1 (41.2)	
WA	Current	10.5 (6.9)	2.5 (18.6)	5.3 (10.1)		6.8 (6.8)	8.5 (5.7)
WA	Future	9.3 (6.2)	2.9 (18.5)	5.8 (8)		4.9 (5.1)	6.5 (11.5)
ESA	Current	10.5 (6.1)	4.3 (4.3)	5.5 (5.9)	8.3 (9.2)	4.7 (22.4)	9.4 (4.2)
ESA	Future	9.9 (4.9)	3.2 (6.1)	4.7 (6.6)	9.4 (7.7)	3.1 (23.7)	7.6 (5.2)
SSA	Current	10.5 (4.7)	2.7 (15.4)	5.3 (8)	8.3 (9.2)	6.6 (4.7)	8.7 (4.5)
SSA	Future	9.6 (3.7)	2.9 (15.9)	5.6 (6.6)	9.4 (7.7)	4.8 (6.4)	6.7 (9.6)

*empty cells indicate absence of crops or areas < 10,000 ha

Table S7. Energy content of each cereal based on different references. Values are in kcal kg^{-1} grain with standard moisture contents of 15.5% for maize, 14% for millet, rice and sorghum, and 13.5% for wheat.

Crop	FAO [22]	INRAE [23]	USDA [24]	Mean
Maize	3008	3245	3521	3258
Millet	2924	3517	3559	3334
Rice	3096	3242	3577	3305
Sorghum	2927	3380	3230	3179
Wheat	2889	3270	3417	3192

Table S8. Percentage increase in yield potential under irrigated conditions compared to rainfed conditions. Data are from the Global Yield Gap Atlas [25].

Crop	Increase of Y_p relative to Y_w (%)
Maize	42
Millet	70
Rice	24
Sorghum	30
Wheat	18
Aggregated based on maize equivalents	44

Supporting Figures

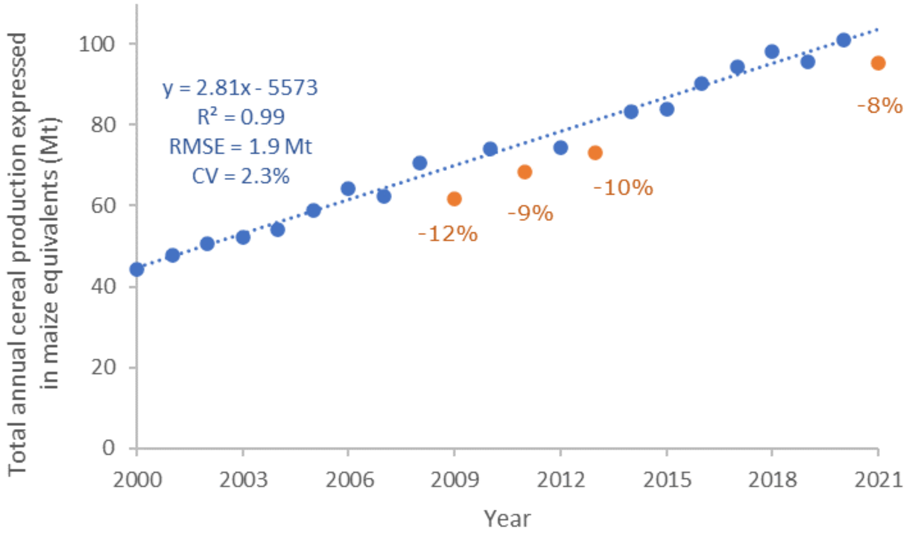

Figure S1. Trend in total annual cereal production (maize, millet, sorghum, rice and wheat) across ten countries in sub-Saharan Africa. The linear regression was applied only to the blue points, which represent years with ‘normal’ weather conditions. The orange points refer to years with ‘extreme’ weather conditions affecting a large part of sub-Saharan Africa such that production deviated from the trend. See Methods in the main manuscript for a detailed explanation and criteria for extreme and normal conditions. Source: FAOSTAT [15].

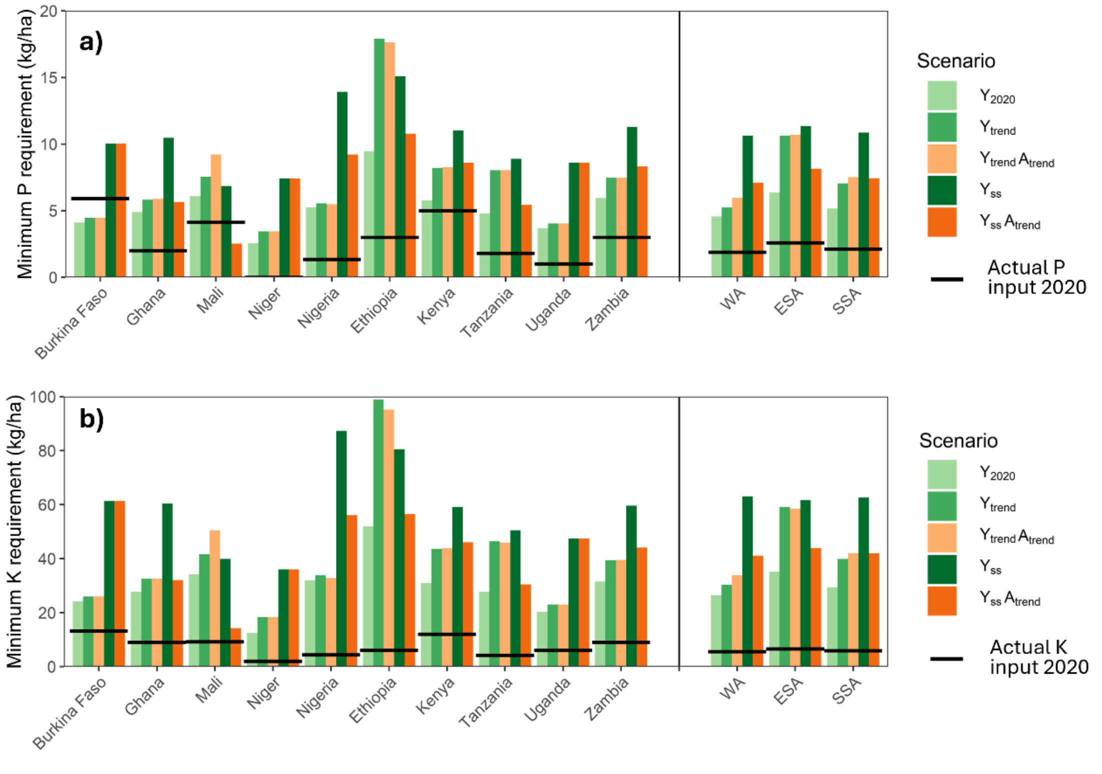

Figure S2. A) Minimum phosphorus (P) and B) potassium (K) requirements for the different countries and regions under different scenarios in 2050 (see Table 1 for specification of all scenarios). Horizontal line represents the actual inputs of P and K in 2020, while the light green bar (Y_{2020}) represents the minimum nutrient requirement for the yields attained in 2020. WA = West Africa; ESA = East and Southern Africa; SSA = sub-Saharan Africa.

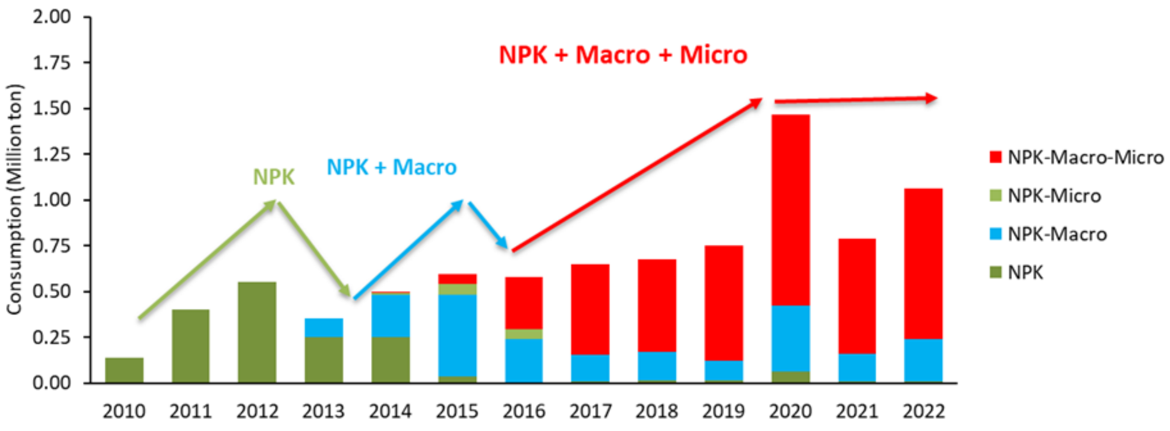

Figure S3. Total amount and types of fertilizers used in Ethiopia from 2010 to 2022. Fertilizers categorized as NPK included at least one of the nutrients nitrogen (N), phosphorus (P), or potassium (K). NPK+Macro indicates the various combinations of primary and secondary macro-nutrients. NPK-micro represents the various combinations of NPK and micro-nutrients. NPK-macro-micro stands for the various combinations of primary and secondary macro- and micro-nutrients. The color of the arrow refers to the type of fertilizer that has been changing mostly in a certain time period. Data are from AfricaFertilizer database [26]. Nutrients added through manure were not considered.

SI References

1. Rosegrant, M.W., et al., *Food and nutrition security under changing climate and socioeconomic conditions.* Global Food Security, 2024. **41** DOI: 10.1016/j.gfs.2024.100755.
2. Wiebe, K., et al., *Climate change impacts on agriculture in 2050 under a range of plausible socioeconomic and emissions scenarios.* Environmental Research Letters, 2015. **10**(8) DOI: 10.1088/1748-9326/10/8/085010.
3. Ignowski, L., et al., *Dietary inadequacy in Tanzania is linked to the rising cost of nutritious foods and consumption of food-away-from-home.* Global Food Security, 2023. **37** DOI: 10.1016/j.gfs.2023.100679.
4. FAO, *The State of Food and Agriculture 2019. Moving forward on food loss and waste reduction*. 2019, Rome.
5. Haile, B., et al., *Nutrition and food security in Burkina Faso diagnostic overview.* 2019, IFPRI. p. 65.
6. Kushitor, S.B., *Food availability and food habits among Ghanaians: Tracking the dietary transition in Ghana.* Nutrition and Health, 2021. **29**(1): p. 157-166 DOI: 10.1177/02601060211066707.
7. Bocoum, I. and S. Dury, *Non parametric and parametric analysis of Engel function for calorie, dietary diversity and food shares in the calories, in rural and urban Mali. .* Journées Jeunes Chercheurs du Département SAE2, Montpellier, FRA, 2009.
8. Akerele, D., *Household Food Expenditure Patterns, Food Nutrient Consumption and Nutritional Vulnerability in Nigeria: Implications for Policy.* Ecol Food Nutr, 2015. **54**(5): p. 546-71 DOI: 10.1080/03670244.2015.1041136.
9. Worku, I.H., et al., *Diet transformation in Africa: the case of Ethiopia.* Agricultural Economics, 2017. **48**(S1): p. 73-86 DOI: 10.1111/agec.12387.
10. Vila-Real, C.P.M., et al., *Nutritional intake and food sources in an adult urban Kenyan population.* Nutr Bull, 2022. **47**(4): p. 423-437 DOI: 10.1111/nbu.12582.
11. Akumu, G., et al., *Dietary patterns in Uganda and their influencing factors: A critical review.* African Journal of Food, Agriculture, Nutrition and Development, 2023. **23**(2): p. 22328-22353 DOI: 10.18697/ajfand.117.22345.
12. Pauw, K. and J. Thurlow, *Agricultural growth, poverty, and nutrition in Tanzania.* Food Policy, 2011. **36**(6): p. 795-804 DOI: 10.1016/j.foodpol.2011.09.002.
13. FAO, *World programme for the census of agriculture 2020, Volume 1 Programme, concepts and definitions*. 2017. p. 190.
14. IFPRI, *Global Spatially-Disaggregated Crop Production Statistics Data for 2020 Version 1.0.0. Harvard Dataverse, V1.* 2024 DOI: doi.org/10.7910/DVN/SWPENT.
15. FAOSTAT. 2024; Available from: https://www.fao.org/faostat/en/#data/QCL.
16. UBOS, *Uganda Annual Agricultural Survey 2018*. 2018, Kampala, Uganda; UBOS. p. 348.
17. UBOS, *Uganda Annual Agricultural Survey 2019*. 2019, Kampala, Uganda; UBOS. p. 414.
18. Alimagham, S., et al., *Climate change impact and adaptation of rainfed cereal crops in sub-Saharan Africa.* European Journal of Agronomy, 2024. **155** DOI: 10.1016/j.eja.2024.127137.
19. van Oort, P.A.J. and S.J. Zwart, *Impacts of climate change on rice production in Africa and causes of simulated yield changes.* Global Change Biology, 2017. **24**(3): p. 1029-1045 DOI: 10.1111/gcb.13967.
20. Ludemann, C.I., et al., *A global FAOSTAT reference database of cropland nutrient budgets and nutrient use efficiency (1961–2020): nitrogen, phosphorus and potassium.* Earth System Science Data, 2024. **16**(1): p. 525-541 DOI: 10.5194/essd-16-525-2024.
21. van Dam, A., *Future crop nutrient requirements of five cereals in Sub-Saharan Africa.* Estimating agronomic nutrient use efficiencies of Sub-Saharan Africa's major cereals. MSc thesis, Wageningen, The Netherlands: Wageningen University, 2020.
22. FAO. 2024; Available from: https://www.fao.org/3/x9892e/X9892e05.htm#TopOfPage.
23. INRAE. 2024; Available from: https://www.feedtables.com/content/sorghum.
24. USDA. 2024; Available from: https://fdc.nal.usda.gov/fdc-app.html#/?query=WHEAT.
25. GYGA. 2024; Available from: https://www.yieldgap.org.
26. AfricaFertilizer. 2024; Available from: https://viz.africafertilizer.org/#/ethiopia/home.
